# The Role of a Seven-Day Physiotherapy Service in Reducing Length of Stay and Improving Cost-Effectiveness in Arthroplasty Surgery

**DOI:** 10.7759/cureus.33951

**Published:** 2023-01-18

**Authors:** Peter F Staunton, Marc C Grant-Freemantle, Eoghan Pomeroy, James Cashman

**Affiliations:** 1 Trauma and Orthopaedics, National Orthopaedic Hospital Cappagh, Dublin, IRL; 2 Orthopaedics, National Orthopaedic Hospital Cappagh, Dublin, IRL

**Keywords:** bed days, efficiency, delay, discharge, arthroplasty

## Abstract

Background

Length of hospital stay post hip and knee arthroplasty is influenced by several factors, including gender, home circumstances and underlying diagnosis. Due to increasing demand for hip and knee arthroplasty, elective units, operating within already stressed healthcare systems, must identify methods of increasing efficiency and capacity. We sought to establish whether the lack of a seven-day inpatient physiotherapy service resulted in an increased hospital length of stay post primary hip and knee arthroplasty.

Methods

One hundred consecutive joint replacements (50 total hip replacements and 50 total knee replacements (TKRs)), performed in our institution from January to February 2020, were assessed. The length of stay for the cohort was analysed, and delays to discharge were identified. T-test was used to analyse the difference in length of stay based on the day of the week the surgery was performed.

Results

The mean length of stay for all primary hip and knee arthroplasties was 3.42 (standard deviation (SD): 1.62) days. Hip and knee arthroplasties performed on a Thursday or Friday had a significantly higher average length of stay than those performed on Monday, Tuesday or Wednesday (3.89 versus 3.02, p=0.006). We calculated that operating a six-day versus seven-day physiotherapy service in our unit cost 318 bed days per year equating to €986,535.

Conclusion

Length of stay post total hip and knee arthroplasty in our unit is significantly affected by the day of the week that surgery is performed. Elective orthopaedic units should consider all means of maximising efficiency and lowering costs given the future challenges in service provision.

## Introduction

Length of hospital stay post hip and knee arthroplasty is influenced by several factors, including gender, home circumstances and underlying diagnosis [1]. These procedures are used to treat arthritis, which has a prevalence of over 900,000 in Ireland [2]. The National Joint Registry data from the United Kingdom indicates that over 200,000 total hip and knee arthroplasties were performed in the UK in 2019 [3]. Published data suggest an increase in demand for these procedures of up to 200% by 2030 [4-6]. This will inevitably place greater stress on healthcare systems, demanding improved efficiency and cost control.

The Organisation for Economic Co-operation and Development (OECD) assesses hospital efficiency by measuring the amount of resources a hospital uses to treat a specific condition, and length of hospital stay is a gross measure of this [7]. The average length of stay for hip and knee arthroplasty has steadily decreased across Irish public hospitals since 2010 when the average post-operative stay was 8.59 days for total hip arthroplasty and 8.79 days for total knee arthroplasty. By 2013, this had improved to 5.65 days for total hip arthroplasty and 5.56 days for total knee arthroplasty, representing a fall of 34% and 37%, respectively [8], and this trend was reflected in UK data [9]. As same-day total hip and knee arthroplasty procedures grow in popularity, the range of length of stay between jurisdictions and between high- and low-volume centres continues to be wide.

Our high-volume centre performs, on average, over 1,400 primary hip and knee arthroplasties per year. Inpatient physiotherapy is provided six days of a week, with Sundays excluded. We sought to establish whether the lack of a seven-day inpatient physiotherapy service impacted length of stay post primary hip and knee arthroplasty.

## Materials and methods

One hundred consecutive primary unilateral joint replacements (50 total hip replacements and 50 total knee replacements (TKRs)), performed in our institution from January to February 2020, were assessed. Cases were identified using our institutional database [10]. Patient demographics including anonymous identifier, age, sex and body mass index (BMI) were used to populate a proforma. An e-chart and physical chart review was performed to identify the time to discharge post-surgery, the time to physiotherapy sign-off post-surgery and the time to discharge post physiotherapy sign-off. Standardised local physiotherapy programmes were delivered to all patients. Where discharge was delayed, the documented reason for the delay was identified. The discharge destination was also recorded. Retrospective data was taken from our institutional database to establish the number of primary total hip and knee arthroplasties performed annually. Figures pertaining to the cost of physiotherapy provision were based on Health Service Executive pay grades [11]. A power study was conducted to establish the numbers needed to power the study at 80%. The required sample size was 70 for a difference of one day in length of stay. Statistical analysis was performed using STATA version 14 (College Station, TX, USA) [12]. T-test was used to analyse the difference in length of stay between days of the week. Single-factor analysis of variance (ANOVA) was used to determine differences in length of stay between surgeons.

## Results

The mean length of stay for all cases was 3.42 (standard deviation (SD): 1.62) days. The total hip arthroplasty mean length of stay was three days, whereas the total knee arthroplasty mean length of stay was 3.86 days. The mean BMI was 30.84 (SD: 5.04) kg/m^2^. Sixteen different fellowship-trained surgeons performed the 100 cases, and there was no statistically significant difference in length of stay for individual surgeons (p=0.477). Of the patients, 40% stayed longer than three days. Of these, 25 (62.5%) had discharge delayed due to failure to achieve physiotherapy goals. Eight (20%) patients had discharge delayed due to medical reasons. Seven (17.5%) patients’ discharges were delayed due to a lack of availability of a convalescence bed in an outside unit. The average length of stay for those undergoing hip or knee arthroplasty surgery from Monday to Wednesday was 3.02 days. When surgery was performed on Thursday or Friday (where the established average length of stay was extended into Sunday), the average length of stay increased to 3.86 days for a Thursday surgery and 3.94 days for a Friday surgery. This showed a significant difference in length of stay for surgeries performed on Monday, Tuesday and Wednesday versus those performed on Thursday and Friday (Table 1). No patient was discharged on Sunday having undergone surgery on a Friday.

**Table 1:** Difference in length of stay depending on the day of surgery

	Monday to Wednesday	Thursday to Friday
Mean	3.02	3.89
Variance	2.52	2.4
Observations	54	46
Pooled variance	2.464	
P (T≤t) two-tail	0.006	

The cost incurred due to the lack of a seven-day physiotherapy service was estimated based on additional bed days. The mean additional length of stay (0.87 days) was multiplied by the average number of joints performed per annum on either a Thursday or Friday (670.22) to calculate the total additional bed days. Failure to reach physiotherapy goals delayed discharge in 62.5% of our extended stay cohort. Additional bed days secondary to delayed physiotherapy goals was calculated based on this percentage. The additional cost of implementing a seven-day physiotherapy service was subtracted from the cost of additional bed days to calculate the estimated potential saving (Table 2).

**Table 2:** Cost savings after the implementation of a seven-day physiotherapy service *46% (Thursday and Friday) of the average number of primary joints performed per year from 2016 to 2019 **Cost of bed days including implants and all overheads (NOHC data) NOHC: National Orthopaedic Hospital Cappagh

Average number of additional bed days		0.8745	
Number of procedures*		670.22	
	Total additional bed days for Thursday/Friday surgery per year		586.10739
	Proportion of additional bed days attributed to delayed rehabilitation		0.625
	Number of additional bed days due to delayed rehabilitation for Thursday/Friday surgery per year		366.32
	Cost per bed day**		€3,100
	Total cost of additional bed days		€1,135,587
Yearly estimated cost of Sunday physiotherapy hours		€45,052	
Cost of additional staff grade physiotherapists × 2		€104,000	
	Total cost of providing Sunday physiotherapy service		€149,052
Total potential savings with Sunday physiotherapy service			€986,535

## Discussion

International healthcare systems face considerable challenges in relation to funding and subsequently to the provision of care [13]. Increasing demand for hip and knee arthroplasty will place significant added pressure on an already stressed system. Increasing efficiency within the existing structures will allow for added capacity and go some way towards addressing these future demands. Large studies assessing risk factors for prolonged length of stay have identified pre-operative factors such as poor Oxford Knee Score (OKS), rheumatoid aetiology and deformity as predictive of prolonged length of stay post-operatively [14]. Our study, however, shows variability in length of stay based on the day of surgery, with an assumption regarding an equal case mix spread across five operative days and 16 different arthroplasty surgeons.

The evidence for physiotherapy in supporting early post-operative mobilisation after hip and knee arthroplasty is well-established and clear [15,16]. Previous studies have shown that failure to provide appropriate physiotherapy post total joint arthroplasty can adversely delay discharge [17]. Our data supports this and suggests that the provision of a seven-day physiotherapy service in our centre, when compared to the existing six-day model, would decrease our average length of stay post hip and knee arthroplasty. The most significant impact would be seen for patients undergoing surgery on a Thursday or Friday as this group appears to be most adversely affected. Importantly, from a long-term outcome point of view, the consequences of delayed physiotherapy are not inconsequential. Where physiotherapy is not commenced as soon as possible, there may be a cumulative knock-on effect with studies showing that early initiation of rehabilitation reduces negative outcomes [18].

Our unit currently provides orthopaedic review, availability of doctors for discharging patients and appropriate nursing cover as part of the weekend service. Based on our cost calculations, the addition of a physiotherapy service on a Sunday has the potential to further streamline efficiency within our hospital system and provide significant cost savings estimated at close to €1,000,000. Operating as the designated National Orthopaedic Hospital, strong consideration needs to be given to addressing all controllable variables that affect length of stay post arthroplasty, and a seven-day physiotherapy service should be the standard of care from a patient care and hospital system point of view.

There are a number of limitations to our study. We examined 100 consecutive primary hip and knee joint replacements over a four-week period. This is a small sample size and provides a small snapshot of total arthroplasty activity in the institution. The conclusions and savings that have been drawn have been extrapolated from the data from this timeframe. While the number of primary arthroplasties performed in this time period does reflect the typical activity of the unit, there could be confounding factors affecting patient length of stay within this timeframe that could potentially influence the results. As well as this, our study assumes an equal case mix spread across five operative days and 16 arthroplasty surgeons, and this variation and assumption could be a potential confounding factor. Cost-effective analysis calculations are based on standardised pay grades and bed cost days in the Irish health service. This may affect the validity and applicability to hospitals that are not high-volume elective centres that deal with both trauma and elective patients and are not within the Irish health service. Delays in reaching physiotherapy goals may not be simply attributed to the lack of a seven-day physiotherapy service. Napier et al., in a similar study, found that the major factor delaying discharge post-TKR was inadequate social support (33%). Their unit, with a seven-day physiotherapy service, still found that 21% of their delayed discharges were due to delays in reaching physiotherapy goals [19]. There are underestimations in our study too as only primary hip and knee arthroplasties were considered in this study. Consideration must also be given to the potential benefit in terms of improved outcomes and subsequent savings on outpatient physiotherapy and extended clinical reviews. There is also potential for the service to provide further savings when revision surgery is accounted for. There are on average 100 revision procedures performed per year in our unit where timely rehabilitation input is also required and may represent a further potential €27,000 saving in bed days.

There have been significant changes over the last 10 years in peri-operative care for hip and knee arthroplasty [20,21]. These changes have reduced length of hospital stay, reduced peri-operative complications and improved efficiency, meaning reduced overall costs [22]. Everyday mechanisms exist within health services to further these improvements, and additional refinement will be required to allow for the expansion of services in line with the expected increase in demand over the next 10 years.

## Conclusions

Length of stay post total hip and knee arthroplasty in our unit is significantly affected by the day of the week that surgery is performed, and surgery later in the week is associated with a longer length of stay. Elective orthopaedic units should consider all means of maximising efficiency and lowering costs given the future challenges in service provision.

## References

[1] Ding ZC, Xu B, Liang ZM, Wang HY, Luo ZY, Zhou ZK (2020). Limited influence of comorbidities on length of stay after total hip arthroplasty: experience of enhanced recovery after surgery. Orthop Surg.

[2] Ireland CSO (2017). Central Statistics Office: Press statement census 2016 results profile 9 - Health, disability and carers. Census.

[3] Board NE (2018). National Joint Registry: 15th Annual Report. https://www.hqip.org.uk/wp-content/uploads/2018/11/NJR-15th-Annual-Report-2018.pdf.

[4] Sloan M, Premkumar A, Sheth NP (2018). Projected volume of primary total joint arthroplasty in the U.S., 2014 to 2030. J Bone Joint Surg Am.

[5] Culliford D, Maskell J, Judge A, Cooper C, Prieto-Alhambra D, Arden NK (2015). Future projections of total hip and knee arthroplasty in the UK: results from the UK Clinical Practice Research Datalink. Osteoarthritis Cartilage.

[6] Ackerman IN, Bohensky MA, Zomer E, Tacey M, Gorelik A, Brand CA, de Steiger R (2019). The projected burden of primary total knee and hip replacement for osteoarthritis in Australia to the year 2030. BMC Musculoskelet Disord.

[7] Lorenzoni L, Marino A (2017). Understanding variations in hospital length of stay and cost. https://www.oecd-ilibrary.org/docserver/ae3a5ce9-en.pdf?expires=1660490966&id=id&accname=guest&checksum=7C473854417B68449EA081B90457399A.

[8] (2015). National Model of Care for Trauma and Orthopaedic Surgery.

[9] Burn E, Edwards CJ, Murray DW (2018). Trends and determinants of length of stay and hospital reimbursement following knee and hip replacement: evidence from linked primary care and NHS hospital records from 1997 to 2014. BMJ Open.

[10] Bluespier. Clanwilliam Group, Droitwich Droitwich (2023). Bluespier. Worcestershire, WR9.

[11] (2023). Health Service Executive: Pay scales. https://www.hse.ie/eng/staff/benefitsservices/pay/.

[12] (2023). Stata Statistical Software. https://www.stata.com/support/faqs/resources/citing-software-documentation-faqs/.

[13] Pabinger C, Lothaller H, Portner N, Geissler A (2018). Projections of hip arthroplasty in OECD countries up to 2050. Hip Int.

[14] Khanna V, Gurava Reddy AV, Daultani D, Sankineani SR, Khanna J, Annapareddy A, Eachampati KK (2019). When can I go home after my knee replacement? Factors affecting the duration of in-hospital stay after knee replacement. Eur J Orthop Surg Traumatol.

[15] Sharma V, Morgan PM, Cheng EY (2009). Factors influencing early rehabilitation after THA: a systematic review. Clin Orthop Relat Res.

[16] Masaracchio M, Hanney WJ, Liu X, Kolber M, Kirker K (2017). Timing of rehabilitation on length of stay and cost in patients with hip or knee joint arthroplasty: a systematic review with meta-analysis. PLoS One.

[17] Panteli M, Habeeb S, McRoberts J, Porteous MJ (2014). Enhanced care for primary hip arthroplasty: factors affecting length of hospital stay. Eur J Orthop Surg Traumatol.

[18] Chiung-Jui Su D, Yuan KS, Weng SF, Hong RB, Wu MP, Wu HM, Chou W (2015). Can early rehabilitation after total hip arthroplasty reduce its major complications and medical expenses? Report from a nationally representative cohort. Biomed Res Int.

[19] Napier RJ, Spence D, Diamond O, O'Brien S, Walsh T, Beverland DE (2013). Modifiable factors delaying early discharge following primary joint arthroplasty. Eur J Orthop Surg Traumatol.

[20] Yanik JM, Bedard NA, Hanley JM, Otero JE, Callaghan JJ, Marsh JL (2018). Rapid recovery total joint arthroplasty is safe, efficient, and cost-effective in the Veterans Administration setting. J Arthroplasty.

[21] Abdel MP, Berry DJ (2019). Current practice trends in primary hip and knee arthroplasties among members of the American Association of Hip and Knee Surgeons: a long-term update. J Arthroplasty.

[22] Sarpong NO, Boddapati V, Herndon CL, Shah RP, Cooper HJ, Geller JA (2019). Trends in length of stay and 30-day complications after total knee arthroplasty: an analysis from 2006 to 2016. J Arthroplasty.

